# Burden of asthma and COPD overlap (ACO) in Taiwan: a nationwide population-based study

**DOI:** 10.1186/s12890-017-0571-7

**Published:** 2018-01-25

**Authors:** Sumitra Shantakumar, Raoh-Fang Pwu, Liesel D’Silva, Keele Wurst, Yao-Wen Kuo, Yen-Yun Yang, Yi-Chen Juan, K. Arnold Chan

**Affiliations:** 1 R&D, Real World Evidence & Epidemiology, GSK, 50 Beach Road, #21-00 Gateway West, Singapore, 189720 Singapore; 2 Health Data Research Center, National Taiwan University, No.33, Linsen South Road, Suite 526, Taipei, 10051 Taiwan; 3 National Respiratory Physician Lead, GSK, 7333 Mississauga Road, Mississauga, ON L5N 6L4 Canada; 4 R&D, Real World Evidence & Epidemiology, GSK, 1250 South Collegeville Road, Collegeville, PA 19426 USA; 5 National Taiwan University Hospital, No.7, Zhongshan S. Road, Zhongzheng District, Taipei City, 10002 Taiwan; 6 Present Address: Ministry of Health and Welfare, Taipei City, Taiwan

**Keywords:** Asthma-chronic obstructive pulmonary disease overlap, Asthma, Chronic obstructive pulmonary disease, Epidemiology, Medical resource utilisation, Taiwan

## Abstract

**Background:**

Patients with symptoms of both asthma and chronic obstructive pulmonary disease (COPD) may be classified with the term asthma-COPD overlap (ACO). ACO is of considerable interest as it is currently poorly characterised and has been associated with worse health outcomes and higher healthcare costs compared with COPD or asthma alone. Patients with ACO in Asia remain poorly described, and there is limited information regarding their resource utilisation compared with patients with asthma or COPD only. This study investigated the characteristics, disease burden and medical resource utilisation of patients with ACO in Taiwan.

**Methods:**

This was a retrospective cohort study of patients identified from National Health Insurance (NHI) claims data in Taiwan in 2009–2011. Patients were classified into incident ACO, COPD or asthma cohorts according to International Classification of Disease, ninth revision, clinical modification codes in claims. Eligible patients were ≥40 years of age with 12 months’ continuous enrolment in the NHI programme pre- and post-index date (date of the first relevant medical claim).

**Results:**

Patients with ACO (*N* = 22,328) and COPD (*N* = 69,648) were older and more likely to be male than those with asthma (*N* = 50,293). Patients with ACO had more comorbidities and exacerbations, with higher medication use: short-acting β_2_-agonist prescriptions ranged from 30.4% of patients (asthma cohort) to 43.6% (ACO cohort), and inhaled corticosteroid/long-acting β_2_-agonist combination prescriptions ranged from 11.1% (COPD cohort) to 35.0% (ACO cohort) in the 12 months following index. Patients with ACO generally had the highest medication costs of any cohort (long-acting muscarinic antagonist costs ranged from $227/patient [asthma cohort] to $349/patient [ACO cohort]); they also experienced more respiratory-related hospital visits than patients with asthma or COPD (mean outpatient/inpatient visits per patient post-index: 9.1/1.9 [ACO cohort] vs 5.7/1.4 [asthma cohort] and 6.4/1.7 [COPD cohort]).

**Conclusions:**

Patients with ACO in Taiwan experience a greater disease burden with greater healthcare resource utilisation, and higher costs, than patients with asthma or COPD alone.

**Electronic supplementary material:**

The online version of this article (10.1186/s12890-017-0571-7) contains supplementary material, which is available to authorized users.

## Background

Patients presenting with overlapping clinical features of asthma and chronic obstructive pulmonary disease (COPD) may be described with the term asthma-COPD overlap (ACO). ACO has been recognised in the Global Initiative for Chronic Obstructive Lung Disease (GOLD) and Global Initiative for Asthma (GINA) guidelines since 2014, when the existence of ACO syndrome (ACOS) was proposed; [1, 2] however, more recent guidance has suggested that, since ACOS cannot be considered to be a single disease entity, the term ACO is more appropriate [3]. ACO is of considerable interest worldwide as it is currently poorly characterised with no clear diagnostic definition, yet it has been associated with worse health outcomes and higher healthcare costs compared with COPD or asthma alone [4, 5]. Most large-scale studies investigating the prevalence of ACO and the characteristics of patients have examined populations outside of Asia [4, 6–11]. Within Asia, several studies have examined Korean, [12–14] Japanese [15–18] and Chinese patients [19, 20]. However, since some studies in Asian patients involved small populations or examined only particular aspects of the disease, patients with ACO in Asia remain poorly described, and there is limited information regarding their resource utilisation compared with patients with asthma or COPD only.

The ACO population in Taiwan is particularly poorly described; one study assessed the prevalence of ACO and compared the incidence of acute respiratory events in patients with ACO and COPD; [21] a second study examined the association between ACO and tuberculosis [22]. However, both studies used only a sample of data from the Taiwanese population and therefore may not capture the full clinical picture. Healthcare in Taiwan is provided through the National Health Insurance (NHI) programme. This single-payer, universal programme provides medical coverage to >99% of the population; [23] therefore, Taiwan is an ideal setting for quantification of the country-level incidence of ACO and its associated healthcare utilisation and costs.

This study aimed to characterise patients with ACO in Taiwan and compare them with patients with asthma or COPD only, examining demographics at diagnosis, disease burden, healthcare and medication resource utilisation and associated costs using recent national administrative health insurance data from the entire Taiwanese population. Additionally, an exploratory analysis of different ACO definitions was performed.

## Methods

### Study design

This was a retrospective cohort study (GSK study number PRJ2356; National Taiwan University grant number 105R104012) of patients meeting criteria for ACO, COPD, or asthma based on NHI claims data for 2009–2011 (patients were followed through 2012). NHI claims were based on International Classification of Disease, ninth revision, clinical modification (ICD-9-CM) codes. Data were obtained through the Health and Welfare Data Science Center, Department of Statistics, Ministry of Health and Welfare in Taiwan.

### Patients

#### Eligibility criteria

Eligible patients were ≥40 years of age with incident NHI disease claims relevant to COPD or asthma (COPD, ICD-9-CM codes: 491 [chronic bronchitis], 492 [emphysema] or 496 [chronic airway obstruction, not elsewhere classified]; asthma, ICD-9-CM code: 493 [asthma]). A disease was considered to be incident when there were no prior relevant claims in the study period, including a look-back period from 2006 to 2008. Patients were also required to have at least 12 months of continuous enrolment in the NHI programme both prior to and following the index date (defined below), and at least three outpatient claims or one inpatient claim in the year that followed. Patients were also required to have at least one dispensing claim for a relevant respiratory treatment (specified below) in the year following the index date.

Exclusion criteria included the presence of conditions (based on ICD-9-CM codes used for claims) related to lung or bronchial developmental anomalies, degenerative processes, bronchiectasis, pulmonary resection or other significant respiratory disorders (excluding cancer) that could interfere with clinical diagnosis of COPD or its natural history (ICD-9-CM codes 490, 494 or 495).

#### Disease cohorts

Three mutually exclusive incident disease cohorts (asthma, COPD and ACO) were defined for analysis based on a combination of ICD-9-CM diagnosis codes and respiratory drug use. The asthma cohort included patients with claims related to asthma (ICD-9-CM code 493) and a prescription for an asthma-specific treatment (specified below), while the COPD cohort included patients with claims related to COPD (ICD-9-CM codes 491, 492 or 496) and a prescription for a COPD-specific treatment. However, patients were excluded from these cohorts if they fulfilled any of the necessary criteria for the ACO cohort, while patients with claims for both asthma and COPD who did not fulfil any of the criteria for the ACO cohort were excluded from the study.

For the ACO cohort, patients conforming to any of four operational definitions of ACO were included. These definitions were not mutually exclusive, but patients conforming to multiple definitions were counted only once in the overall ACO cohort. The four operational definitions explored were as follows:

1. ACO-I: patients with a claim related to chronic obstructive asthma (ICD-9-CM code: 493.2×) at the index date and at least 3 outpatient claims and/or at least 1 inpatient claim with this code in the year post-index, with or without additional asthma or COPD codes;
2. ACO-II: patients with ICD-9-CM codes for both asthma and COPD in a single claim at the index date and at least 3 outpatient claims and/or at least 1 inpatient claim with codes for both asthma and COPD in the year post-index;
3. ACO-III: patients with incident COPD, as defined for the COPD cohort above, with at least 3 outpatient claims and/or at least 1 inpatient claim for asthma in the 12 months pre-index;
4. ACO-IV: patients with incident asthma, as defined for the asthma cohort above, with at least 3 outpatient claims and/or at least 1 inpatient claim for COPD in the 12 months pre-index.

#### Index date definition

**Fig. 1:**
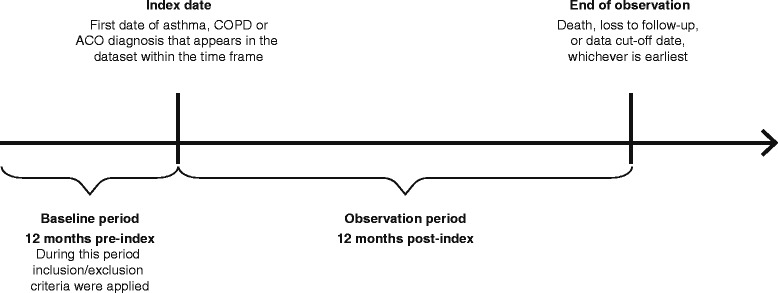
Study design. ACO, asthma-COPD overlap; COPD, chronic obstructive pulmonary disease

The index date for the COPD and asthma cohorts was the date of the first relevant medical claim in the study time frame (encompassing the look-back period from 2006 to 2008) (Fig. 1). For the ACO cohort, the index date was the date of the first medical claim for the related diagnosis: the date of the first claim with the 493.2× ICD-9-CM code [ACO-I definition]; the date of the first claim with both asthma and COPD codes [ACO-II]; the date of the first claim with a COPD code [ACO-III]; or the date of the first claim with an asthma code [ACO-IV]. Where patients met the criteria for more than one ACO definition with different possible index dates, the earlier index date was used.

### Relevant respiratory treatments

Relevant respiratory treatments for COPD included: short- or long-acting muscarinic antagonists (SAMAs or LAMAs), short- or long-acting β_2_-agonists (SABAs or LABAs), inhaled corticosteroids (ICS), ICS/LABA combinations, systemic corticosteroids, xanthine or systemic β_2_-agonists. Relevant treatments for asthma or ACO comprised all of these classes, plus leukotriene receptor antagonists (LTRAs) or anti-immunoglobulin E (anti-IgE) therapy. Each prescription was assumed to be of 30 days’ duration with the exception of systemic corticosteroids, which were classified as being prescribed for either 14 days or fewer, or more than 14 days. Monotherapies were those that did not overlap with prescriptions of other therapies, while multiple therapies in combination or separate inhalers had to be prescribed together or overlap by one day to be considered part of a combination.

### Outcomes

The following parameters were characterised and compared between cohorts: patient demographics and comorbidities (at index date); treatment with respiratory medication and medication costs (in the 12 months post-index); incidence of exacerbations (post-index); and healthcare utilisation (post-index, including: frequency of outpatient, inpatient, emergency room [ER] and intensive care unit [ICU] hospital visits; and use of X-rays, computed tomography [CT] and pulmonary function tests).

Three definitions of exacerbations were employed based on ICD-9-CM codes, and the requirement for a prescription of systemic corticosteroids for <14 days. Definition 1 required: ICD-9-CM codes 491, 492 or 496 for COPD; ICD-9-CM code 493 for asthma; and a combination of these events for ACO. Definition 2 was the same as for Definition 1, with the addition of ICD-9-CM codes 480–486 for the COPD and asthma cohorts. Definition 3 required: ICD-9-CM code 491.21 for COPD; ICD-9-CM code 493.92 for asthma; and a combination of these events for ACO.

All cost data were measured in New Taiwan Dollars and converted to US dollars (USD) using the exchange rate at the end of the data collection period (31 December, 2012) [24].

### Statistical analysis

Differences between variables were assessed using the chi-squared test for categorical variables, and the student’s t-test for continuous variables. All tests were two-sided and *p*-values < 0.05 were considered statistically significant. Analyses were performed using SAS software version 9.4 (SAS Institute, Inc., Cary, NC).

## Results

### Patient demographics

**Fig. 2:**
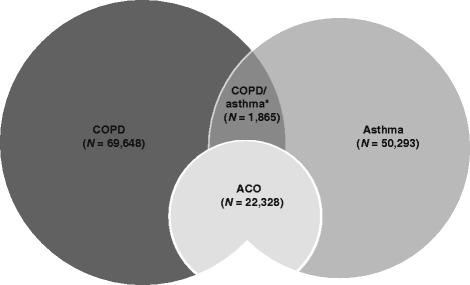
Diagram of the four cohorts identified in the study. *Patients with claims for both COPD and asthma not meeting any of four pre-defined criteria for ACO. ACO, asthma-COPD overlap; COPD, chronic obstructive pulmonary disease

Of 480,900 patients with claims involving COPD codes, 69,648 patients met the criteria for the ‘COPD only’ cohort (Additional file 1: Figure S1). Of 621,670 patients with claims involving asthma codes, 50,293 patients met the criteria for the ‘asthma only’ cohort (Additional file 1: Figure S2). The ACO cohort comprised 22,328 patients (ACO-I, 7711 patients; ACO-II, 5572 patients; ACO-III, 5458 patients; ACO-IV, 8765 patients; some patients met the criteria for more than one definition) (Additional file 1: Figure S3). Using this figure, it can be estimated that the incidence of ACO among adults of ≥40 years of age in Taiwan during the study period (2009–2011) is approximately 7.0 per 10,000, based on the average mid-year populations in this period. There were 1865 patients with claims with both asthma and COPD codes, but who did not meet any of the four pre-defined criteria for ACO (the COPD/asthma cohort) and were excluded from the analysis. The overall distribution of the patient cohorts is shown in Fig. 2.

Patients in the ACO and COPD cohorts had similar mean ages and were more likely to be male, while patients in the asthma cohort were younger and more likely to be female (Table 1). Patients in the ACO cohort had the highest mean Charlson comorbidity index (CCI) score (*p* < 0.0001 vs asthma and COPD), while patients with asthma had the lowest score. In particular, patients with ACO had a significantly higher presence of cardiovascular comorbidities (cerebrovascular disease, congestive heart failure and hypertension) than patients with COPD or asthma (*p* < 0.0001, all comparisons).

**Table 1:** Demographics of the ACO, COPD and asthma cohorts at index date

Characteristics	ACO cohort (*N* = 22,328)	COPD cohort (*N* = 69,648)	Asthma cohort (*N* = 50,293)	*p*-value (ACO vs COPD)	*p*-value (ACO vs asthma)	*p*-value (3 groups)
Gender, *n* (%)						
Male	13,477 (60.4)	45,165 (64.9)	19,499 (38.8)	< 0.0001	< 0.0001	< 0.0001
Age						
Mean (SD), years	68.7 (13.3)	68.1 (13.4)	60.6 (13.3)	< 0.0001	< 0.0001	< 0.0001
Socioeconomic status (monthly income), *n* (%)						
High: ≥$1035	5118 (22.9)	15,144 (21.7)	16,013 (31.8)	< 0.0001	< 0.0001	< 0.0001
Medium: $690–$1035	9110 (40.8)	29,832 (42.8)	19,922 (39.6)			
Low: <$690	7558 (33.9)	22,430 (32.2)	13,838 (27.5)			
Unknown	542 (2.4)	2242 (3.2)	520 (1.0)			
Charlson comorbidity index, mean (SD)	0.91 (1.49)	0.85 (1.57)	0.51 (1.17)	< 0.0001	< 0.0001	< 0.0001
Comorbidities^a^, *n* (%)						
Anaemia	1136 (5.1)	3086 (4.4)	1144 (2.3)	< 0.0001	< 0.0001	< 0.0001
Anxiety	1576 (7.1)	3612 (5.2)	3351 (6.7)	< 0.0001	0.0505	< 0.0001
Cancer	1272 (5.7)	4200 (6.0)	1764 (3.5)	0.0668	< 0.0001	< 0.0001
Cerebrovascular disease	3337 (15.0)	9372 (13.5)	2846 (5.7)	< 0.0001	< 0.0001	< 0.0001
Congestive heart failure	1566 (7.0)	2817 (4.0)	1267 (2.5)	< 0.0001	< 0.0001	< 0.0001
Dementia	1283 (5.8)	3577 (5.1)	977 (1.9)	0.0004	< 0.0001	< 0.0001
Diabetes mellitus	3442 (15.4)	10,386 (14.9)	5947 (11.8)	0.0670	< 0.0001	< 0.0001
Hypertension	8950 (40.1)	22,136 (31.8)	13,802 (27.4)	< 0.0001	< 0.0001	< 0.0001
Peptic ulcer	2698 (12.1)	6119 (8.8)	3393 (6.8)	< 0.0001	< 0.0001	< 0.0001

Differences between variables were assessed using the chi-squared test (categorical variables) or the student’s t-test (continuous variables). All tests were two-sided and *p*-values < 0.05 were considered statistically significant

*ACO* asthma-COPD overlap, *COPD* chronic obstructive pulmonary disease, *SD* standard deviation

^a^Only comorbidities occurring at rates >5% in any cohort are presented

Across the four groups with different ACO definitions (Additional file 1: Table S1) the percentage of male patients ranged from 52.5% (ACO-III) to 66.2% (ACO-I) versus 60.4% for the ACO cohort overall; the mean age ranged from 65.8 years (ACO-II) to 72.0 years (ACO-IV) versus 68.7 years for the ACO cohort overall; and the mean CCI score ranged from 0.66 (ACO-II) to 1.13 (ACO-IV) versus 0.91 for the ACO cohort overall.

### Respiratory-related medication use and costs

**Fig. 3:**
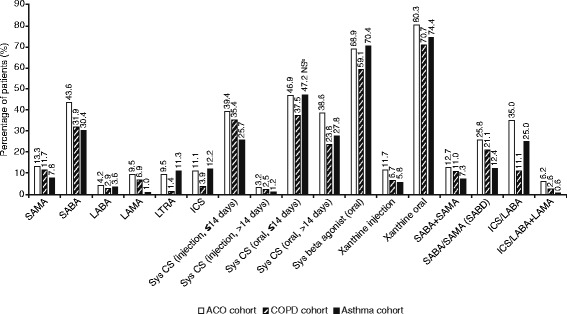
Respiratory-related medication use in the 12 months following the index date^a^. ^a^Only medications prescribed to >1% of patients in at least two cohorts are presented. ^b^All comparisons with ACO were statistically significant (*p* ≤ 0.001) except for the use of oral systemic corticosteroids (≤14 days) between the ACO and COPD cohorts (*p* = 0.500). ACO, asthma-COPD overlap; COPD, chronic obstructive pulmonary disease; CS, corticosteroid; ICS, inhaled corticosteroid; IgE, immunoglobulin E; LABA, long-acting β_2_-agonist; LAMA, long-acting muscarinic antagonist; LTRA, leukotriene receptor antagonist; NS, not significant; SABA, short-acting β_2_-agonist; SABD, short-acting bronchodilator; SAMA, short-acting muscarinic antagonist; Sys, systemic

In all cohorts, the most commonly prescribed medication in the 12 months post-index was oral xanthine, followed by oral systemic β_2_-agonists and oral systemic corticosteroids (≤14 days) (Fig. 3). SABAs were among the most commonly prescribed medications (30.4–43.6% of patients), while SAMAs were less frequently prescribed (7.8–13.3% of patients). All short-acting therapies (SABAs, SAMAs and SABA/SAMA combinations) were more commonly prescribed than long-acting bronchodilator monotherapies (LAMAs or LABAs) in all cohorts. ICS/LABA combinations were also prescribed more commonly than LAMAs or LABAs alone. Oral systemic corticosteroids and ≤14-day courses of injected systemic corticosteroids were prescribed more frequently than ICS.

For most medications, the prescription rate was highest in the ACO cohort (Fig. 3); however, prescription of ICS, LTRAs and oral systemic β_2_-agonists was higher in patients with asthma than patients with ACO (*p* < 0.0001) or COPD. Some differences in prescription frequencies between cohorts were considerable: ICS and ICS/LABA combinations were prescribed approximately three times more frequently in patients with ACO than COPD (ICS: 11.1% vs 3.9%; ICS/LABA: 35.0% vs 11.1%), while LTRAs were prescribed over six times more frequently (9.5% vs 1.4%). LAMA monotherapy and ICS/LABA + LAMA open triple therapy were prescribed almost ten times more frequently in patients with ACO than asthma (LAMA: 9.5% vs 1.0%; ICS/LABA + LAMA: 6.2% vs 0.6%). In patients with COPD, LAMA monotherapy was prescribed approximately seven times more frequently as in patients with asthma (6.9% vs 1.0%; Fig. 3).

The total cost of each medication in the 12 months post-index was generally highest in the COPD cohort, although the asthma cohort had the highest total cost for anti-IgE, ICS, LTRA and ICS/LABA medications (Table 2). However, when assessed as the mean cost per patient, the costs of most medications were highest in the ACO cohort.

**Table 2:** Respiratory-related medication costs in the 12 months post-index date

Medication type^a^	ACO cohort (*N* = 22,328)			COPD only (*N* = 69,648)			Asthma only (*N* = 50,293)		
	Total cost (USD)	Number of patients	Mean cost/person (USD)	Total cost (USD)	Number of patients	Mean cost/person (USD)	Total cost (USD)	Number of patients	Mean cost/person (USD)
Anti-IgE	28,993	< 5^b^		2842	< 5^b^		61,397	6	10,233
ICS	132,777	2482	53	116,536	2710	43	283,766	6136	46
LTRA	329,286	2119	155	81,646	993	82	738,900	5659	131
LABA	16,242	944	17	35,013	2023	17	16,909	1832	9
ICS/LABA	2,224,844	7820	285	1,582,052	7732	205	2,465,128	12,579	196
LAMA	742,647	2125	349	1,497,123	4826	310	110,641	488	227
SABA	276,888	9731	28	461,267	22,227	21	222,497	15,299	15
SAMA	86,336	2974	29	181,629	8179	22	54,971	3900	14
SABA/SAMA (SABD)	319,419	5758	55	653,839	14,720	44	181,868	6228	29
Systemic β_2_-agonist injection	6	20	0.3	34	62	1	15	48	0.3
Systemic β_2_-agonist oral	241,131	15,384	16	444,653	41,179	11	194,135	35,419	5
Systemic corticosteroids injection (≤14 days)	223,498	8794	25	480,051	24,662	19	197,142	12,936	15
Systemic corticosteroids injection (>14 days)	43,054	719	60	96,799	1745	55	33,618	619	54
Systemic corticosteroids oral (≤14 days)	18,951	10,480	2	34,385	26,126	1	30,325	23,742	1
Systemic corticosteroids oral (>14 days)	62,043	8620	7	97,912	16,564	6	61,374	13,994	4
Xanthine injection	3469	2611	1	4517	4629	1	2102	2894	1
Xanthine oral	295,758	17,934	16	483,469	49,230	10	217,083	37,405	6
Xanthine unknown	2	6	0.3	0.4	8	0.1	0	5	0.0

Differences between variables were assessed using the chi-squared test (categorical variables) or the student’s t-test (continuous variables). All tests were two-sided and *p*-values < 0.05 were considered statistically significant

*ACO* asthma-COPD overlap, *COPD* chronic obstructive pulmonary disease, *ICS* inhaled corticosteroid, *IgE* immunoglobulin E, *LABA* long-acting β_2_-agonist, *LAMA* long-acting muscarinic antagonist, *LTRA* leukotriene receptor antagonist, *SABA* short-acting β_2_-agonist, *SABD* short-acting bronchodilator, *SAMA* short-acting muscarinic antagonist, *USD* US dollars

^a^Categories were not mutually exclusive

^b^Numbers <5 could not be retrieved from the Health and Welfare Data Science Center

When comparing the different medications, ICS/LABA therapy contributed the most to the total medication costs in all cohorts. This was followed by LAMA monotherapy in the ACO and COPD cohorts, while the second greatest contributor in the asthma cohort was LTRA therapy. Other medications were minor contributors to the overall medication cost for each cohort, contributing <11% of the total. Despite the high use of oral xanthine and oral systemic β_2_-agonists, their costs contributed <8% of the total medication cost in any cohort. Although anti-IgE therapy had the highest cost per patient in all cohorts, it was rarely prescribed (≤6 patients in any cohort). Of the other medications, LAMA monotherapy had the highest cost per patient in all cohorts, followed by ICS/LABA therapy.

As in the overall ACO cohort, oral xanthine and oral systemic β_2_-agonists were the most commonly prescribed medications in each of the groups with different ACO definitions, while prescription rates of ICS, LAMAs, LTRAs and ICS/LABA + LAMA combinations were among the lowest of the medications (Additional file 1: Table S2).

There were significant differences in the use of most medications between the groups with different ACO definitions (*p*-values for each ACO group vs the overall ACO cohort were < 0.05 for ≥8 of the 15 medications prescribed to >5% of patients in the ACO cohort; Additional file 1: Table S2). However, the magnitudes of the differences in medication use between the different ACO groups were <12% for all medications except for ICS/LABA, which had a difference of 16.3% between the ACO-I and ACO-II groups (31.4% vs 47.7% of patients). The medications with the next largest differences between groups were systemic corticosteroid injections for ≤14 days (prescription rates ranged from 33.3% [ACO-II] to 44.9% [ACO-IV]), and SABAs (prescription rates ranged from 41.5% [ACO-II] to 49.2% [ACO-IV]) (Additional file 1: Table S2).

### All-cause medical utilisation

In the 12 months post-index, patients with ACO had a significantly higher mean number of all-cause outpatient visits, ER visits and ICU admissions than patients with asthma or COPD (*p* < 0.05, all comparisons) (Additional file 1: Table S3). However, patients with COPD had the highest mean number of inpatient visits (*p* < 0.05, all comparisons). Pulmonary function tests were used most commonly in the ACO cohort, while use of CT was highest in the COPD cohort. X-ray use was similar in the ACO and COPD cohorts and higher than in the asthma cohort (Additional file 1: Table S3).

### Respiratory-related medical utilisation

In the 12 months post-index, patients in the ACO cohort had the highest mean numbers of respiratory-related outpatient and ER visits (Table 3). Patients with ACO also had a significantly higher mean number of hospitalisations and ICU admissions than patients with asthma or COPD (*p* < 0.0001). A higher percentage of patients with ACO experienced all types of hospital visit than patients with asthma or COPD (data not shown).

**Table 3:** Respiratory-related medical utilisation in the 12 months post-index date

Utilisation type	ACO cohort (*N* = 22,328)	COPD cohort (*N* = 69,648)	Asthma cohort (*N* = 50,293)	*p*-value (ACO vs COPD)	*p*-value (ACO vs asthma)	*p*-value (3 groups)
Medical utilisation (outpatient)						
Mean number of outpatient visits (SD)	9.14 (7.48)	6.43 (5.39)	5.68 (4.36)	< 0.0001	< 0.0001	< 0.0001
Mean number of ER visits (SD)	2.01 (3.20)	1.43 (1.00)	1.42 (1.22)	< 0.0001	< 0.0001	< 0.0001
Medical utilisation (inpatient)						
Mean number of inpatient visits (SD)	1.93 (1.54)	1.68 (1.38)	1.38 (0.95)	< 0.0001	< 0.0001	< 0.0001
Mean number of ICU admissions (SD)	1.31 (0.70)	1.20 (0.53)	1.14 (0.42)	< 0.0001	< 0.0001	< 0.0001
X-rays, *n* (%)	11,390 (51.0)	30,044 (43.1)	13,046 (25.9)	< 0.0001	< 0.0001	< 0.0001
Computed tomography, *n* (%)	2010 (9.0)	6293 (9.0)	1737 (3.5)	0.8800	< 0.0001	< 0.0001
Pulmonary function tests, *n* (%)	4724 (21.2)	10,157 (14.6)	7243 (14.4)	< 0.0001	< 0.0001	< 0.0001
Exacerbations, *n* (%)						
Event 1^a^	7883 (35.3)	12,934 (18.6)	14,583 (29.0)	< 0.0001	< 0.0001	< 0.0001
Event 2^b^	2207 (9.9)	3658 (5.3)	1876 (3.7)	< 0.0001	< 0.0001	< 0.0001
Event 3^c^	3083 (13.8)	5068 (7.3)	2577 (5.1)	< 0.0001	< 0.0001	< 0.0001

Differences between variables were assessed using the chi-squared test (categorical variables) or the student’s t-test (continuous variables). All tests were two-sided and *p*-values < 0.05 were considered statistically significant

*ACO* asthma-COPD overlap, *COPD* chronic obstructive pulmonary disease, *ER* emergency room, *ICD-9-CM* International Classification of Disease, ninth revision, clinical modification, *ICU* intensive care unit, *SD* standard deviation

^a^Event 1 for:

• COPD: visits with ICD-9 code of 491, 492 or 496 with prescription of systemic corticosteroids for <14 days

• Asthma: visits with ICD-9 code of 493 with prescription of systemic corticosteroids for <14 days

• ACO: combination of COPD and asthma event

^b^Event 2 for:

• COPD: visits with ICD-9 code of 491, 492 or 496, and 480–486, with prescription of systemic corticosteroids for <14 days

• Asthma: visits with ICD-9 code of 493 and 480–486, with prescription of systemic corticosteroids for <14 days

• ACO: combination of COPD and asthma event

^c^Event 3 for:

• COPD: visits with ICD-9 code of 491.21 with prescription of systemic corticosteroids for <14 days

• Asthma: visits with ICD-9-CM code of 493.92 with prescription of systemic corticosteroids for <14 days

• ACO: combination of COPD and asthma event

Use of X-ray and pulmonary function tests was significantly higher in the ACO cohort (X-ray: 51.0%; pulmonary function tests: 21.2%) than in the asthma (X-ray: 25.9%; pulmonary function tests: 14.4%) or COPD cohorts (X-ray: 43.1%; pulmonary function tests: 14.6%) (Table 3). Use of CT was similar between the ACO and COPD cohorts (both 9.0% [*p* = 0.8800]) and higher than in the asthma cohort (3.5%).

Of the groups with different ACO definitions, patients in the ACO-IV group generally had the highest resource use, with the highest mean number of outpatient visits and the highest use of X-ray and CT scans (Additional file 1: Table S4). Patients in the ACO-II group had the lowest resource use with the lowest percentages of patients experiencing ER visits, hospitalisations and ICU admissions (data not shown).

### Exacerbations

In the 12 months post-index, more patients with ACO experienced exacerbations (35.3%) than patients with asthma (29.0%, *p* < 0.0001 vs ACO) or COPD (18.6%, *p* < 0.0001 vs ACO) based on Definition 1. The same pattern was observed for the other two definitions (Table 3). The higher rate of exacerbations in the ACO cohort was reflected in the higher use of injected systemic corticosteroids (≤14 days) in this cohort; however, the use of oral systemic corticosteroids (≤14 days) was similar between the ACO and COPD cohorts. More exacerbations required outpatient than inpatient treatment in each cohort (outpatient vs inpatient visits: ACO: 28.0% vs 15.2%; asthma: 26.7% vs 3.7%; COPD: 12.1% vs 8.8%). Patients with ACO had considerably higher rates of exacerbations requiring ER visits (8.2% vs 2.7% for the COPD cohort [*p* < 0.0001] and vs 2.3% for the asthma cohort [*p* < 0.0001]) and ICU treatment (4.3% vs 2.7% for the COPD cohort [*p* < 0.0001] and vs 0.5% for the asthma cohort [*p* < 0.0001]) than patients with COPD or asthma. The same pattern was observed for the other two definitions of exacerbations.

Of the groups with different ACO definitions, the percentage of patients experiencing exacerbations in the 12 months post-index was highest in the ACO-IV group (38.5% using Definition 1) and lowest in the ACO-II group (33.4% using Definition 1) (Additional file 1: Table S4).

## Discussion

Overall, these findings suggest that patients with ACO in Taiwan experience a considerably higher disease burden than patients with asthma or COPD alone, as evidenced by patterns of healthcare and medication utilisation. These results are consistent with findings from studies in other Asian countries (Korea, [12, 13] Japan [16] and China [19]) and Western countries [4–6, 8, 9].

Several studies have investigated the prevalence of ACO, with estimates ranging from 1.6% to 4.5% across studies in Italy, Latin America, South Korea, and the United States [25]. However, few estimates of incidence have been made; one study reported an estimated incidence of ACO in a Danish population sample of 0.64 per 1000 person-years, [26] while another study reported an estimated incidence in the Ontario population of 1.9 per 1000 people in 2012 [27]. As with prevalence estimates, some degree of variation might be expected due to differences in populations and definitions of ACO used between studies. However, further research is required to corroborate the estimated incidence in this study.

The similarity in age and gender between patients with ACO and COPD, and the lower age and higher proportion of females in patients with asthma, observed here are consistent with studies in Korea [14] and Japan [18]. The mean age and gender distribution in patients with ACO here are also consistent with smaller studies in Taiwan; [21, 22] however, studies outside Asia mostly found that patients with ACO were younger [10, 28] and more likely to be female [8, 10, 11] than those with COPD. These differences may relate to the requirement for patients in this study to be ≥40 years of age, thereby pre-selecting an older population.

The higher presence of comorbidities in patients with ACO than COPD or asthma found here is consistent with studies in Korea, [12] Spain [11] and the USA, [10] although other studies have reported a similar or lower presence of comorbidities in patients with ACO compared with COPD [14, 18]. These inconsistencies may be related to varying procedures for recruiting and categorising patients, and varying inclusion and exclusion criteria [25].

The higher medication use and higher associated costs in patients with ACO compared with asthma or COPD has been observed in several other studies [8, 10, 12]. The higher use of LTRA and ICS in patients with asthma compared with patients with COPD, and higher use of LAMA-containing agents in patients with COPD compared with patients with asthma, are consistent with the GINA [29, 30] and GOLD [31, 32] guideline recommendations available during the study and the differences in asthma and COPD phenotypes.

The high use of xanthine oral therapy in all cohorts is perhaps surprising given its reported poorer efficacy compared with alternative therapies, higher risk of side effects [33–35] and resultant low priority in the GINA [29, 30] and GOLD [31, 32] guidelines available during this study (this is maintained in the current guidelines [3, 36]). Inhaled β_2_-agonists have a quicker onset of action and reduced risk of side effects compared with their oral counterparts, [29–32] but here, use of oral β_2_-agonists was higher than inhaled β_2_-agonists in all cohorts. This is consistent with studies of COPD therapy in the Taiwanese population [37] and other Asian countries, [38] which attributed the higher relative use of oral bronchodilators to lower costs and less clinical evidence being required for their prescription; patients may also find oral medication easier to take. European studies suggest that the use of xanthines and oral bronchodilators for COPD treatment is considerably lower than reported in Asia, while use of LAMA, ICS and ICS/LABA combinations tends to be higher [39–42].

Patients in the ACO cohort were often treated with short-term (≤14 days) systemic corticosteroids, SABAs and ICS/LABA combinations. Although guidelines specific to ACO were not available at the time of this study, more recent GOLD and GINA guidelines recommended ICS therapy with the possible addition of a LABA and/or LAMA [3, 43]. The use of short-term corticosteroids and SABAs observed here may relate to the higher exacerbation frequency in patients with ACO, which could potentially be reduced with longer-term use of guideline-recommended therapy.

The higher rate of hospital visits for patients with ACO compared with COPD was consistent with studies in Korea [12, 13] and China [19]. Reported rates of hospital visits may vary between Asian and other countries as specialists are more commonly located in public hospitals in Asia, while more outpatient or private care options may be available in other countries due to variations in reimbursement systems. An Asian study examining medical costs found that patients with ACO had higher medical costs than patients with COPD, [12] as seen here. Outside Asia, several studies report similar findings, with patients with ACO having a higher presence of comorbidities and exacerbations than patients with COPD or asthma (the higher rate of exacerbations in patients with ACO found here is consistent with other studies within Asia [19] and elsewhere [4, 8, 11, 28]). Patients with ACO experienced more hospitalisations and ER visits [6, 8, 10, 44] than patients with asthma or COPD, with higher resulting medical costs [10, 44, 45].

As there is no consensus on a formal definition of ACO, an exploratory analysis was performed to examine the impact of different definitions, using four different algorithms to define patients with ACO (ACO groups I-IV). Out of these different groups, patients in the ACO-IV group (incident asthma and a previous history of COPD) tended to have the highest resource use. Due to their history of COPD, these patients tended to be older and may have progressed further in the course of their disease (they also had a higher presence of comorbidities) than patients in the ACO-I and ACO-II groups, who were newly diagnosed with chronic obstructive asthma, or with concurrent asthma and COPD. These newly diagnosed patients had lower resource use in the 12 months post-index, suggesting that earlier diagnosis and intervention may reduce medical costs; alternatively, as these patients were younger, they may have progressed less far in the course of their disease and so would not be directly comparable with the older patients. These differences demonstrate that varying definitions of ACO can affect patient outcome measures; ultimately, greater consistency between definitions used in different studies would enable clearer comparisons to be made and overall trends to be identified. The lack of a clear definition makes it challenging to characterise patients and their outcomes, but also demonstrates the heterogeneity of patients described as having ACO, suggesting a spectrum of pathophysiology between COPD and asthma that cannot necessarily be split into well-defined groups [46, 47]. Further follow-up of these different patient groups could provide more evidence to assess the value of earlier diagnosis and intervention, and to compare the values of the different ACO definitions.

A possible consequence of the higher disease burden observed in patients with ACO compared with those with asthma and COPD alone is that patients with ACO may have a poorer long-term prognosis. However, studies assessing long-term outcomes in patients with ACO have shown mixed results: a study in the USA reported a higher mortality risk in patients with ACO compared with patients with asthma or COPD, [7] while an Italian study reported that the long-term prognosis of patients with ACO was poorer than that of patients with asthma, but similar to that of patients with COPD [48]. Studies in Spain [49] and Sweden [50] reported lower mortality rates in patients with ACO than in patients with COPD alone, while a Canadian study reported a lower all-cause mortality rate, but a higher respiratory-cause mortality rate, in patients with ACO compared with COPD or asthma [51]. Similarly mixed results have been reported in Asian populations, although fewer studies have been undertaken: studies in China [52] and Japan [18, 53] have reported lower mortality rates in patients with ACO compared with COPD, while a study in Korea reported a higher mortality rate in patients with ACO [13]. To the best of our knowledge, the long-term prognosis and mortality rates of patients with ACO have not been assessed in the Taiwanese population; this would be an interesting extension to the current study. The conflicting nature of these results can be at least partly attributed to the considerable variations between studies in characteristics and sizes of patient populations, type of data sources used, factors adjusted for in models and follow-up period duration, as well as the definition of ACO employed. The differences in long-term prognosis between COPD, asthma and ACO cannot therefore be established; further research with consistent, systematic methodology is required to confirm whether the higher disease burden in patients with ACO translates to a poorer long-term prognosis.

A strength of this study was that the patients were from a population-based data source without any sampling, and that the data source had near-total population coverage; therefore, the data are representative of the entire Taiwanese population. A limitation was the potential risk of patient misclassification, as classification was dependent on ICD-9-CM codes and how accurately the codes were applied, rather than spirometric or other clinical data, which were unavailable. Patients may have received both asthma and COPD-related ICD-9-CM codes in claims to access certain medications, rather than due to a definitive classification of ACO. Data on smoking status were also unavailable for analysis, so it was not possible to take smoking history into account when assigning disease classifications. There appears to be a growing consensus that tobacco exposure is a requirement for a classification of ACO, while patients with asthma and non-fully reversible airflow obstruction, but without a history of tobacco exposure, should be classified as having obstructive asthma and not ACO [54–56]. Without smoking history data, it was not possible to distinguish between such patients in this study. A further limitation of this study was the requirement for patients to have at least three relevant outpatient claims or one relevant inpatient claim in the year following index date, which reflects a relatively high level of patient contact with healthcare providers that may not be generalisable to other countries and healthcare systems.

## Conclusions

Patients with ACO in Taiwan experience a higher disease burden than patients with asthma or COPD alone, as evidenced by their higher presence of comorbidities and exacerbations, and their increased healthcare and medication utilisation. Healthcare costs associated with ACO also appear to be higher than those for asthma or COPD alone. Further research into how to define and diagnose patients with ACO will help to further characterise this important and diverse patient population.

## Additional file

Additional file 1: Figure S1. Patient flow diagram for the COPD cohort. **Figure S2.** Patient flow diagram for the asthma cohort. **Figure S3.** Patient flow diagram for the ACO cohort and groups with different ACO definitions. **Table S1.** Characteristics of the ACO cohort and groups with different ACO definitions at index date. **Table S2.** Respiratory-related medication use in the 12 months post-index date for the ACO cohort and groups with different ACO definitions. **Table S3.** All-cause medical utilisation in the asthma, COPD and ACO cohorts in the 12 months post-index date. **Table S4.** Respiratory-related medical utilisation in the 12 months post-index date for the ACO cohort and groups with different ACO definitions. (PDF 2324 kb)

## Abbreviations

ACO: Asthma COPD overlap
anti-IgE: Anti-immunoglobulin E
CCI: Charlson comorbidity index
COPD: Chronic obstructive pulmonary disease
CT: Computed tomography
ER: Emergency room
GINA: Global Initiative for Asthma
GOLD: Global Initiative for Chronic Obstructive Lung Disease
ICD-9-CM: International Classification of Disease, ninth revision, clinical modification
ICS: Inhaled corticosteroid
ICU: Intensive care unit
LABA: Long-acting β_2_-agonist
LAMA: Long-acting muscarinic antagonist
LTRA: Leukotriene receptor antagonist
NHI: National Health Insurance
NS: Not significant
SABA: Short-acting β_2_-agonist
SABD: Short-acting bronchodilator
SAMA: Short-acting muscarinic antagonist
SD: Standard deviation
USD: US dollars
