# Supplementary material for: Burden of asthma and COPD overlap (ACO) in Taiwan: a nationwide population-based study
Source: BMC Pulm Med. 2018 Jan 25;18:16. doi: 10.1186/s12890-017-0571-7 (PMC5784537; doi:10.1186/s12890-017-0571-7)
Supplement: Additional file 1: Figure S1. — Patient flow diagram for the COPD cohort. Figure S2. Patient flow diagram for the asthma cohort. Figure S3. Patient flow diagram for the ACO cohort and groups with different ACO definitions. Table S1. Characteristics of the ACO cohort and groups with different ACO definitions at index date. Table S2. Respiratory-related medication use in the 12 months post-index date for the ACO cohort and groups with different ACO definitions. Table S3. All-cause medical utilisation in the asthma, COPD and ACO cohorts in the 12 months post-index date. Table S4. Respiratory-related medical utilisation in the 12 months post-index date for the ACO cohort and groups with different ACO definitions. (PDF 2324 kb) [file 12890_2017_571_MOESM1_ESM.pdf]

Burden of Asthma and COPD Overlap (ACO) in Taiwan: a Nationwide Population-Based Study – Supplementary Material

Supplementary Figure 1. Patient flow diagram for the COPD cohort

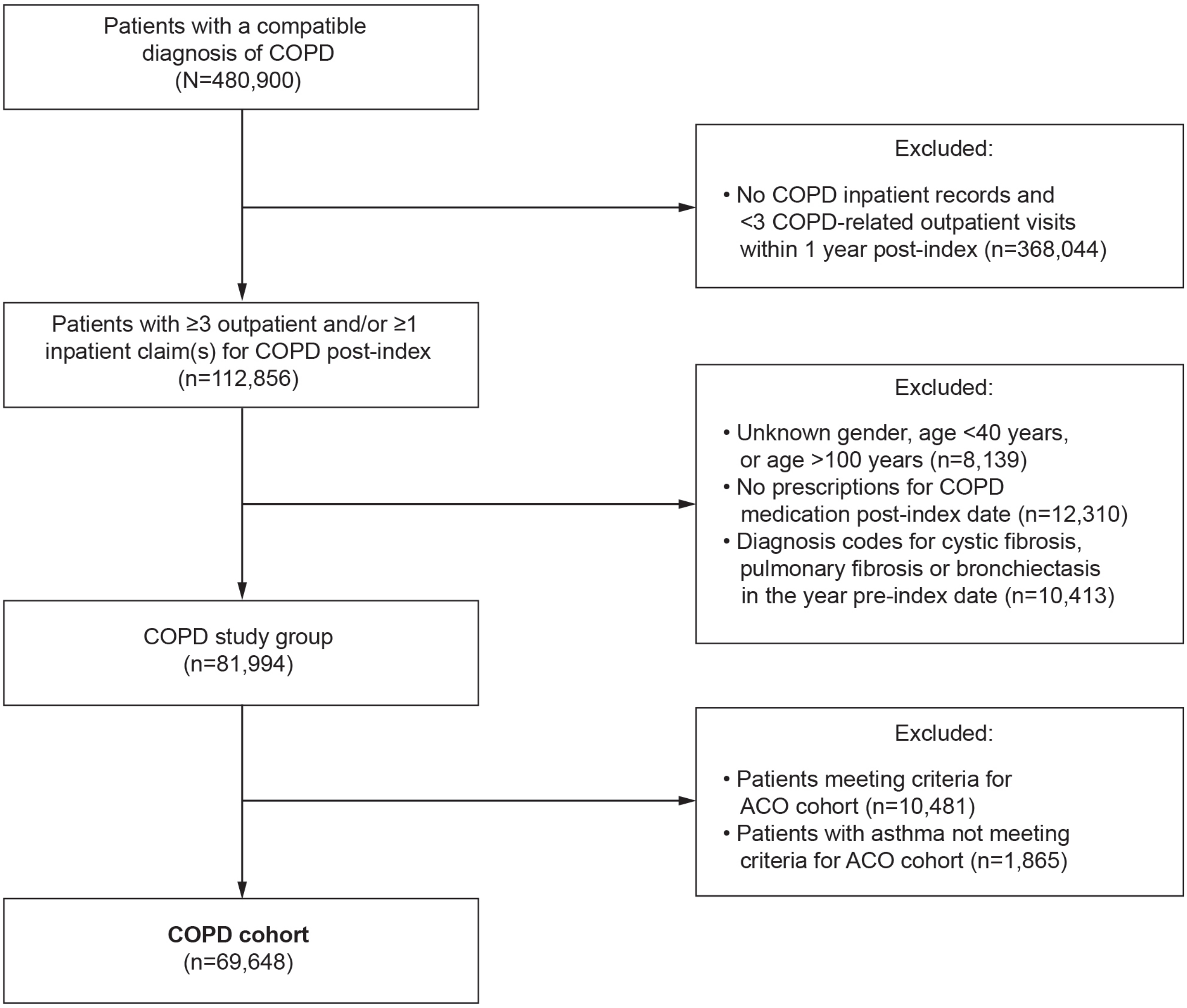

ACO, asthma-COPD overlap; COPD, chronic obstructive pulmonary disease.

Supplementary Figure 2. Patient flow diagram for the asthma cohort

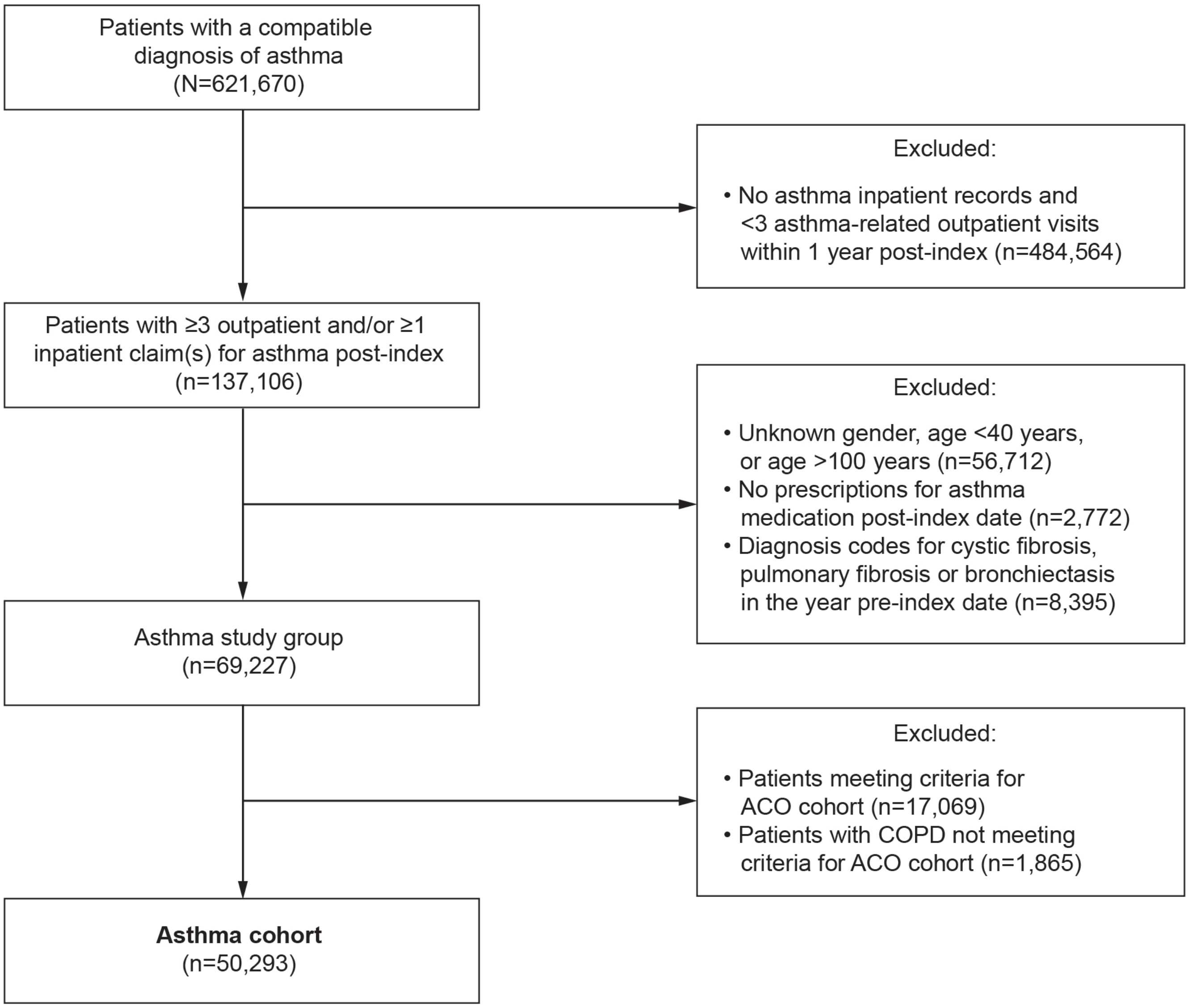

ACO, asthma-COPD overlap; COPD, chronic obstructive pulmonary disease.

Supplementary Figure 3. Patient flow diagram for the ACO cohort and groups with different ACO definitions

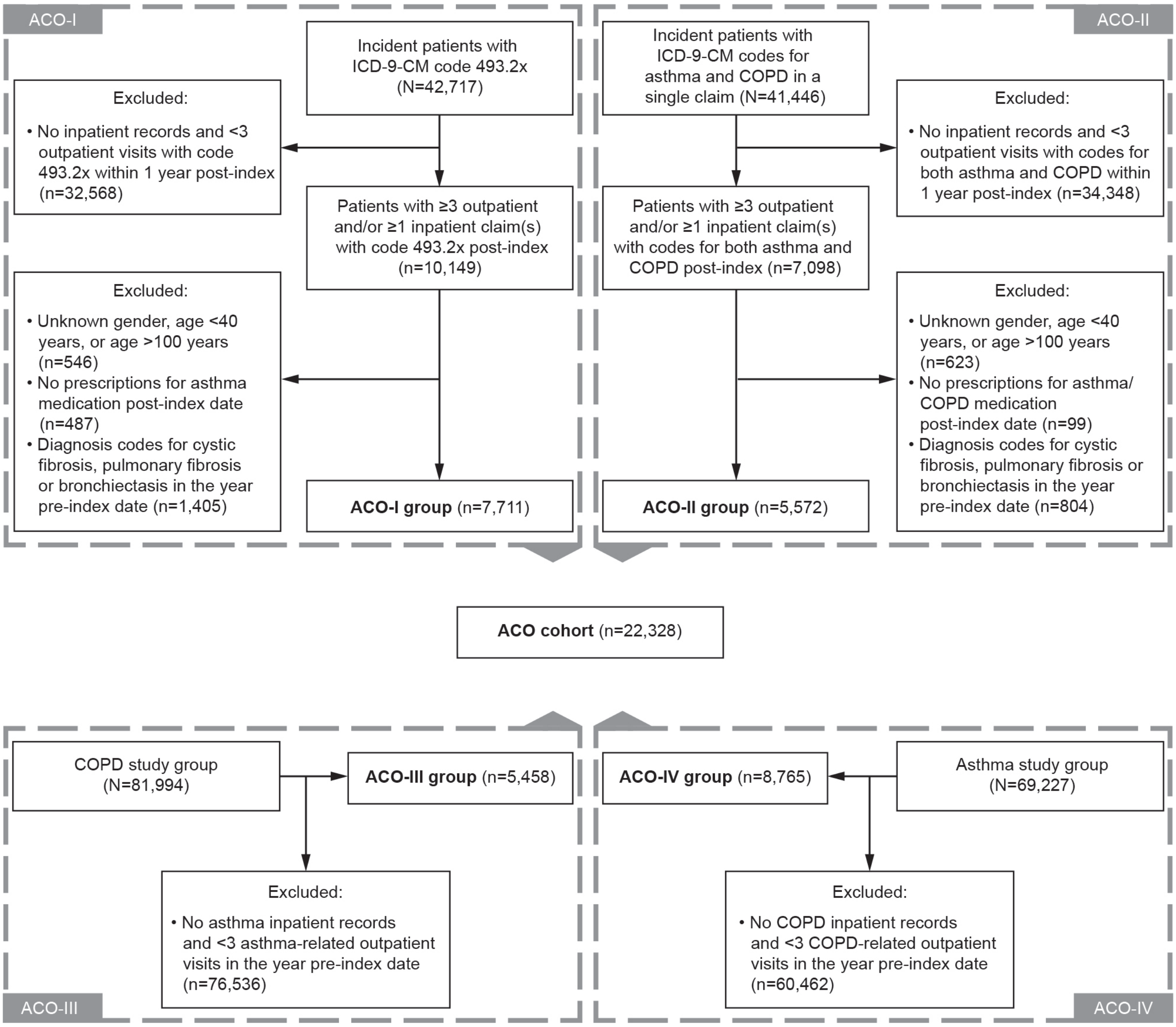

ACO, asthma-COPD overlap; COPD, chronic obstructive pulmonary disease; ICD-9-CM, International Classification of Diseases, ninth revision, clinical modification.

Supplementary Table 1. Characteristics of the ACO cohort and groups with different ACO definitions at index date

Characteristics	ACO cohort (N=22,328)	ACO-I group (n=7,711)	ACO-II group (n=5,572)	ACO-III group (n=5,458)	ACO-IV group (n=8,765)	p-value (ACO cohort vs ACO-I)	p-value (ACO cohort vs ACO-II)	p-value (ACO cohort vs ACO-III)	p-value (ACO cohort vs ACO-IV)	p-value (5 groups)
**Gender, n (%)**										
Male	13,477 (60.4)	5,105 (66.2)	3,499 (61.9)	2,864 (52.5)	5,676 (64.8)	<0.0001	0.0353	<0.0001	<0.0001	<0.0001
**Age**										
Mean (SD), years	68.73 (13.26)	69.67 (13.24)	65.77 (12.92)	66.21 (13.00)	72.00 (12.70)	<0.0001	<0.0001	<0.0001	<0.0001	<0.0001
**Socioeconomic status (monthly income), n (%)**										
High: ≥$1,035	5,118 (22.9)	1,669 (21.6)	1,473 (26.4)	1,370 (25.1)	1,691 (19.3)	0.0079	<0.0001	<0.0001	<0.0001	<0.0001
Medium: $690–$1,035	9,110 (40.8)	3,113 (40.4)	2,195 (39.4)	2,286 (41.9)	3,528 (40.3)					
Low: <$690	7,558 (33.9)	2,706 (35.1)	1,838 (33.0)	1,680 (30.8)	3,270 (37.3)					
Unknown	542 (2.4)	223 (2.9)	66 (1.2)	122 (2.2)	276 (3.2)					
**Charlson co-morbidity index, mean (SD)**	0.91 (1.49)	0.94 (1.53)	0.66 (1.23)	0.84 (1.49)	1.13 (1.59)	0.0771	<0.0001	0.0018	<0.0001	<0.0001

ACO, asthma-chronic obstructive pulmonary disease overlap; SD, standard deviation.

Supplementary Table 2. Respiratory-related medication use in the 12 months post-index date for the ACO cohort and groups with different ACO definitions

Medication type[a], n (%)	ACO cohort (N=22,328)	ACO-I group (n=7,711)	ACO-II group (n=5,572)	ACO-III group (n=5,458)	ACO-IV group (n=8,765)	p-value (ACO cohort vs ACO-I)	p-value (ACO cohort vs ACO-II)	p-value (ACO cohort vs ACO-III)	p-value (ACO cohort vs ACO-IV)	p-value (5 groups)
ICS	2,482 (11.1)	685 (8.9)	745 (13.4)	605 (11.1)	1,120 (12.8)	<0.0001	<0.0001	0.9472	<0.0001	<0.0001
LAMA	2,125 (9.5)	764 (9.9)	702 (12.6)	543 (10.0)	900 (10.3)	0.3157	<0.0001	0.3321	0.0444	<0.0001
LTRA	2,119 (9.5)	653 (8.5)	715 (12.8)	505 (9.3)	772 (8.8)	0.0075	<0.0001	0.5901	0.0622	<0.0001
ICS/LABA	7,820 (35.0)	2,422 (31.4)	2,659 (47.7)	2,046 (37.5)	2,990 (34.1)	<0.0001	<0.0001	0.0007	0.1294	<0.0001
ICS/LABA+LAMA	1,373 (6.2)	445 (5.8)	524 (9.4)	356 (6.5)	611 (7.0)	0.2298	<0.0001	0.3061	0.0076	<0.0001
SABA	9,731 (43.6)	3,290 (42.7)	2,312 (41.5)	2,496 (45.7)	4,310 (49.2)	0.1618	0.0049	0.0041	<0.0001	<0.0001
SAMA	2,974 (13.3)	957 (12.4)	636 (11.4)	815 (14.9)	1,408 (16.1)	0.0413	0.0002	0.0019	<0.0001	<0.0001
SABA/SAMA (SABD)	5,758 (25.8)	1,976 (25.6)	1,260 (22.6)	1,509 (27.7)	2,585 (29.5)	0.7784	<0.0001	0.0051	<0.0001	<0.0001
SABA+SAMA	2,836 (12.7)	914 (11.9)	608 (10.9)	780 (14.3)	1,345 (15.4)	0.0520	0.0003	0.0018	<0.0001	<0.0001
Systemic beta agonist (oral)	15,384 (68.9)	5,110 (66.3)	3,800 (68.2)	3,949 (72.4)	6,167 (70.4)	<0.0001	0.3119	<0.0001	0.0121	<0.0001
Systemic corticosteroid (injection, ≤14 days)	8,794 (39.4)	3,040 (39.4)	1,855 (33.3)	2,222 (40.7)	3,935 (44.9)	0.9522	<0.0001	0.0728	<0.0001	<0.0001
Systemic corticosteroid (oral, ≤14 days)	10,480 (46.9)	3,465 (44.9)	2,644 (47.5)	2,723 (49.9)	4,205 (48.0)	0.0024	0.4908	<0.0001	0.0989	<0.0001
Systemic corticosteroid (oral, >14 days)	8,620 (38.6)	2,924 (37.9)	2,333 (41.9)	2,235 (41.0)	3,519 (40.2)	0.2854	<0.0001	0.0015	0.0121	<0.0001
Xanthine (injection)	2,611 (11.7)	995 (12.9)	581 (10.4)	656 (12.0)	1,146 (13.1)	0.0048	0.0079	0.5037	0.0008	<0.0001
Xanthine (oral)	17,934 (80.3)	6,023 (78.1)	4,620 (82.9)	4,511 (82.7)	7,083 (80.8)	<0.0001	<0.0001	<0.0001	0.3275	<0.0001

[a]Only medications prescribed to >5% of patients in the ACO cohort are presented. Categories were not mutually exclusive.

ACO, asthma-chronic obstructive pulmonary disease overlap; ICS, inhaled corticosteroid; LABA, long-acting beta$_2$-agonist; LAMA, long-acting muscarinic antagonist; LTRA, leukotriene receptor antagonist; SABA, short-acting beta$_2$-agonist; SABD, short-acting bronchodilator; SAMA, short-acting muscarinic antagonist.

Supplementary Table 3. All-cause medical utilisation in the asthma, COPD and ACO cohorts in the 12 months post-index date

Utilisation type	ACO cohort (N=22,328)	COPD cohort (N=69,648)	Asthma cohort (N=50,293)	p-value (ACO vs COPD)	p-value (ACO vs asthma)	p-value (3 groups)
**Medical utilisation (outpatient)**						
Mean number of outpatient visits (SD)	29.87 (20.55)	26.93 (18.98)	26.39 (18.16)	<0.0001	<0.0001	<0.0001
Mean number of ER visits (SD)	2.47 (3.26)	2.35 (2.96)	1.98 (2.37)	0.0011	<0.0001	<0.0001
**Medical utilisation (inpatient)**						
Mean number of inpatient visits (SD)	2.39 (2.04)	2.45 (2.14)	1.92 (1.70)	0.0190	<0.0001	<0.0001
Mean number of ICU admissions (SD)	1.44 (0.83)	1.36 (0.74)	1.27 (0.66)	<0.0001	<0.0001	<0.0001
**X-rays, n (%)**	15,651 (70.1)	48,737 (70.0)	25,696 (51.1)	0.7342	<0.0001	<0.0001
**Computed tomography, n (%)**	5,202 (23.3)	19,431 (27.9)	7,607 (15.1)	<0.0001	<0.0001	<0.0001
**Pulmonary function tests, n (%)**	5,298 (23.7)	12,437 (17.9)	8,686 (17.3)	<0.0001	<0.0001	<0.0001
**Exacerbations, n (%)**						
Event 1[a]	7,883 (35.3)	12,934 (18.6)	14,583 (29.0)	<0.0001	<0.0001	<0.0001
Event 2[b]	2,207 (9.9)	3,658 (5.3)	1,876 (3.7)	<0.0001	<0.0001	<0.0001
Event 3[c]	3,083 (13.8)	5,068 (7.3)	2,577 (5.1)	<0.0001	<0.0001	<0.0001

[a]Event 1 for:

- COPD: visits with ICD-9 code of 491, 492 or 496 with prescription of systemic corticosteroids for <14 days
- Asthma: visits with ICD-9 code of 493 with prescription of systemic corticosteroids for <14 days
- ACO: combination of COPD and asthma event

[b]Event 2 for:

- COPD: visits with ICD-9 code of 491, 492 or 496, and 480–486, with prescription of systemic corticosteroids for <14 days
- Asthma: visits with ICD-9 code of 493 and 480–486, with prescription of systemic corticosteroids for <14 days
- ACO: combination of COPD and asthma event

[c]Event 3 for:

- COPD: visits with ICD-9 code of 491.21 with prescription of systemic corticosteroids for <14 days
- Asthma: visits with ICD-9-CM code of 493.92 with prescription of systemic corticosteroids for <14 days
- ACO: combination of COPD and asthma event

ACO, asthma-COPD overlap; COPD, chronic obstructive pulmonary disease; ER, emergency room; ICD-9, International Classification of Disease, ninth revision, clinical modification; ICS, inhaled corticosteroids; ICU, intensive care unit; SD, standard deviation.

Supplementary Table 4. Respiratory-related medical utilisation in the 12 months post-index date for the ACO cohort and groups with different ACO definitions

Utilisation type	ACO cohort (N=22,328)	ACO-I group (n=7,711)	ACO-II group (n=5,572)	ACO-III group (n=5,458)	ACO-IV group (n=8,765)	p-value (ACO cohort vs ACO-I)	p-value (ACO cohort vs ACO-II)	p-value (ACO cohort vs ACO-III)	p-value (ACO cohort vs ACO-IV)	p-value (5 groups)
**Medical utilisation (outpatient)**										
Mean number of outpatient visits (SD)	9.14 (7.48)	9.40 (7.61)	9.08 (7.46)	9.17 (7.29)	10.06 (8.14)	0.0094	0.6176	0.7676	<0.0001	<0.0001
Mean number of ER visits (SD)	2.01 (3.20)	2.10 (3.34)	2.43 (4.65)	2.05 (3.88)	1.90 (2.06)	0.4471	0.0051	0.7642	0.2307	0.0139
**Medical utilisation (inpatient)**										
Mean number of inpatient visits (SD)	1.93 (1.54)	2.03 (1.64)	1.92 (1.59)	1.75 (1.41)	2.08 (1.65)	0.0181	0.9547	0.0002	<0.0001	<0.0001
Mean number of ICU admissions (SD)	1.31 (0.70)	1.39 (0.80)	1.34 (0.71)	1.19 (0.53)	1.34 (0.75)	0.0366	0.6169	0.0067	0.3245	0.0043
**X-rays, n (%)**	11,390 (51.0)	4,016 (52.1)	2,757 (49.5)	2,794 (51.2)	4,922 (56.2)	0.1053	0.0406	0.8128	<0.0001	<0.0001
**Computed Tomography, n (%)**	2,010 (9.0)	702 (9.1)	475 (8.5)	510 (9.3)	878 (10.0)	0.7881	0.2631	0.4304	0.0055	0.0233
**Pulmonary function tests, n (%)**	4,724 (21.2)	1,487 (19.3)	1,559 (28.0)	1,259 (23.1)	1,740 (19.9)	0.0005	<0.0001	0.0021	0.0107	<0.0001
**Exacerbations, n (%)**										
Event 1[a]	7,883 (35.3)	2,619 (34.0)	1,863 (33.4)	2,015 (36.9)	3,374 (38.5)	0.0333	0.0088	0.0257	<0.0001	<0.0001
Event 2[b]	2,207 (9.9)	813 (10.5)	419 (7.5)	526 (9.6)	1,115 (12.7)	0.0971	<0.0001	0.5825	<0.0001	<0.0001
Event 3[c]	3,083 (13.8)	1,014 (13.2)	714 (12.8)	817 (15.0)	1,475 (16.8)	0.1468	0.0530	0.0269	<0.0001	<0.0001

[a]Event 1 for:

- COPD: visits with ICD-9 code of 491, 492 or 496 with prescription of systemic corticosteroids for <14 days
- Asthma: visits with ICD-9 code of 493 with prescription of systemic corticosteroids for <14 days
- ACO: combination of COPD and asthma event

[b]Event 2 for:

- COPD: visits with ICD-9 code of 491, 492 or 496, and 480–486, with prescription of systemic corticosteroids for <14 days
- Asthma: visits with ICD-9 code of 493 and 480–486, with prescription of systemic corticosteroids for <14 days
- ACO: combination of COPD and asthma event

[c]Event 3 for:

- COPD: visits with ICD-9 code of 491.21 with prescription of systemic corticosteroids for <14 days
- Asthma: visits with ICD-9-CM code of 493.92 with prescription of systemic corticosteroids for <14 days
- ACO: combination of COPD and asthma event

ACO, asthma-COPD overlap; COPD, chronic obstructive pulmonary disease; ER, emergency room; ICD-9, International Classification of Disease, ninth revision, clinical modification; ICS, inhaled corticosteroids; ICU, intensive care unit; SD, standard deviation.
